# Mechanisms and Therapeutic Implications of GSK-3 in Treating Neurodegeneration

**DOI:** 10.3390/cells10020262

**Published:** 2021-01-29

**Authors:** Ido Rippin, Hagit Eldar-Finkelman

**Affiliations:** Department of Human Molecular Genetics and Biochemistry, Sackler School of Medicine, Tel Aviv University, Tel Aviv 6997801, Israel; idorip.ny@gmail.com

**Keywords:** GSK-3, neurodegeneration, microtubules, mTOR, autophagy, lysosome, mitochondria, GSK-3 inhibitors

## Abstract

Neurodegenerative disorders are spreading worldwide and are one of the greatest threats to public health. There is currently no adequate therapy for these disorders, and therefore there is an urgent need to accelerate the discovery and development of effective treatments. Although neurodegenerative disorders are broad ranging and highly complex, they may share overlapping mechanisms, and thus potentially manifest common targets for therapeutic interventions. Glycogen synthase kinase-3 (GSK-3) is now acknowledged to be a central player in regulating mood behavior, cognitive functions, and neuron viability. Indeed, many targets controlled by GSK-3 are critically involved in progressing neuron deterioration and disease pathogenesis. In this review, we focus on three pathways that represent prominent mechanisms linking GSK-3 with neurodegenerative disorders: cytoskeleton organization, the mammalian target of rapamycin (mTOR)/autophagy axis, and mitochondria. We also consider the challenges and opportunities in the development of GSK-3 inhibitors for treating neurodegeneration.

## 1. GSK-3—A Story of Two Isozymes

Glycogen synthase kinase-3 (GSK-3) is a highly conserved protein serine/threonine kinase that plays a central role in a wide variety of cellular processes concerned with coordinating catabolic and anabolic pathways and regulating cellular fate and cell growth. GSK-3 targeted phosphorylation typically inhibits the activity of the substrate, leading to attenuation of the signaling pathway. GSK-3 thus functions as a suppressor of hormone/growth factor-induced signaling cascades. For example, GSK-3 inhibits insulin signaling through the phosphorylation of glycogen synthase and the insulin receptor substrates, IRS-1/IRS-2, where the former leads to inhibition of glycogen synthesis, and the latter inhibits insulin receptor tyrosine kinase activity [1,2,3,4]. GSK-3 also inhibits the canonical Wnt signaling pathway through phosphorylation of β-catenin, which de-stabilizes the protein, leading to subsequent degradation in the proteasome [5,6]. In addition, GSK-3 phosphorylates a variety of transcription factors including Nuclear Factor of Activated T-Cells, NFAT [7], heat shock factor-1 [8], cAMP response element binding protein, CREB, [9], and nuclear factor-kappa B, NF-κB [10], to inhibit gene expression. The unique properties of GSK-3 may explain its involvement in such a wide variety of biological processes: Unlike most of protein kinases, GSK-3 is active under “basal” conditions and is inhibited when cells are stimulated. The substrate recognition is also unusual as it typically requires pre-phosphorylation of the substrate by another “priming kinase” [1,11]. This unique feature adds additional levels of regulation because the ability of GSK to phosphorylate a substrate is conditionally dependent upon the activation of the priming kinase, and that may be controlled by various factors including cell type and cellular context. It should be noted, however, that unprimed substrates had been reported, such as β-catenin, or presenilin-1, as demonstrated by using a GSK-3 mutant that cannot interact with primed substrates [12,13,14]. Finally, the versatility of GSK-3 also relies on its broad range of substrate, including a predicted number over 500 substrates and about 100 “physiological substrates” that are related to diverse cellular functions [15,16]. Another important feature of GSK-3 is the existence of two isozymes, GSK-3α and GSK-3β coded by two different genes [17], and a spliced variant of GSK-3β (GSK-3β2) containing a 13 amino acid insert has been described [18]. The GSK-3β2 variant is enriched in neurons and shows lower in vitro activity as compared to GSK-3β [19]. GSK-3 isozymes exhibit both similar and distinct functions. In some cases, the isozymes fulfil non-redundant physiological functions, but in others, there is a possibility of compensation. The GSK-3 isozymes share 97% identity in their catalytic domains, but there are significant differences at the N–and C-terminal domains [17]. Notably, GSK-3α has been largely overlooked in favor of studies with GSK-3β, although roles for GSK-3α in cellular regulation and diseases pathogenesis have recently been described. From the evolutionary perspective, the α and β isozymes split from a common precursor approximately at the time of emergence of vertebrates, and both genes are highly conserved in fish, amphibians, reptiles, and mammals [20]. An interesting finding is that the α gene is missing in birds. Although the initial findings were based on the available draft genome of three species, namely, chickens, domestic turkeys, and zebra-finches [20], searching the updated genomic data confirms the general selective loss of GSK-3α in the avian species (results from our laboratory).

The question of whether or not each GSK-3 isozyme possesses distinct functions has been addressed in many intense studies. One possible cause of the differences between the isozymes could stem from their distinct distribution in the brain, where GSK3α is especially abundant in the hippocampus, cerebral cortex, striatum, and cerebellum, while GSK3β is expressed in nearly all brain regions [21]. Another option could be that the differences are due to their distinct phosphorylation pattern of substrates. Thus, specific deletion of each of the GSK3 isozymes in the brain produced a distinct substrate phosphorylation pattern [19]. For example, phosphorylation of Collapsin response mediated proteins, CRMP2 and CRMP4 at phosphorylation sites Thr 509, Thr 514 and Ser 518 was not detectable in cortex lacking GSK3β but was normal in cortex lacking GSK-3α, and phosphorylation of tau at Thr 231, Thr 235, and Se 396 was predominantly catalyzed by GSK-3β [19], although there may also be redundant activity of the GSK-3 isozymes for other substrates such as β-catenin [22].

In the following section, we summarize the results obtained by genetic manipulations of the GSK-3 isozymes, focusing on phenotypes and processes related to the brain and the nervous system. A significant difference between the isozymes is clearly observed in embryonic development: while loss of GSK-3β is lethal, due to liver degeneration and impaired heart development [23,24,25], GSK-3α null mice are viable [26]. However, it is apparently more complex to distinguish the roles played by the individual GSK-3 isozymes in adult neurons. The brains of GSK-3α null mice are smaller, and the mice exhibit more aggressive behavior, reduced exploratory activity, and reduced social interaction than normal controls [27]. The GSK-3α null mice also have a shortened lifespan that is associated with age-related pathology related to cardiac dysfunction, early onset of sarcopenia, and cellular senescence [28]. Selective loss of GSK-3α in neurons has also been shown to alter neuronal architecture and behavior activity [29]. With respect to pathological conditions, knock-down of GSK3α, but not GSK3β, ameliorated amyloid plaque loads and memory deficits in an Alzheimer’s disease (AD) mouse model [30]. In contrast, manipulation of GSK-3β expression resulted in alterations in neuronal structure, mood behavior, and cognitive functions. Selective loss of GSK-3β in the forebrain pyramidal neurons produced anxiolytic (reduced anxiety) and pro-social effects [31], and loss of GSK-3β but not GSK-3α in GABAergic neurons, reversed gamma oscillation deficits and cognitive dysfunction in an NMDA hypofunction model related to schizophrenia [32]. Another “behavior” study reported that GSK-3β heterozygous mice exhibit reduced exploratory and anxiety behavior [33,34]. The impact of GSK-3β on neuronal structure was further demonstrated in cortical and hippocampal neurons where selective deletion of GSK-3β reduced dendritic spine stability and attenuated excitatory synaptic transmission [35]. Finally, overexpression of GSK3β reduced brain size in transgenic mice [36].

Conditional deletion of both GSK-3 isozymes further highlighted the prominent role of GSK-3 in regulating brain architecture and behavior skills. Genetic elimination of both GSK-3 isozymes by shRNA reduced axon growth, while localized inhibition of both isozymes at the distal axon resulted in axon elongation [37]. Conditional deletion of GSK-3α and GSK-3β in astrocytes resulted in a larger brain with an increased number of astrocytes [38]. These animals showed aberrant anxiety and altered social behavior [38]. Specific deletion of GSK-3 isozymes in new born cortical neurons, disrupted dendritic orientation and radial migration (moving neurons to a different brain layer) in all areas of the cortex and hippocampus [39]. Finally, deletion of both GSK-3 isozymes in neuronal progenitors resulted in a massive proliferation of cells and prevented progenitor differentiation [40].

The observation that birds lack GSK-3α provides an opportunity to distinguish the specific roles of GSK-3β. Inhibition of brain GSK-3β in a zebra finch model altered singing behavior and reduced neurogenesis in certain regions of the ventricular zone [41]. The results suggested that GSK-3α may be the major tau kinase in the adult brain, as levels of phosphorylated tau (at GSK-3 phosphorylation site) in the bird’s brain were largely reduced as compared to that of found in the mouse brain, a phenomenon that was also recapitulated in the brain of GSK-3α KO mice [20]. As high levels of tau phosphorylation was found in the bird’s embryo, it was further suggested that GSK-3β may be the dominant tau kinase during embryonic development [20]. Interestingly, overexpression of GSK-3β resulted in increased tau phosphorylation in the adult mouse brain [36,42]. Thus, it seems that GSK-3α may be the preferred tau kinase in adult; nevertheless, GSK-3β may become a “more dominant” tau kinase in pathological conditions [36,42].

An interesting alternative model for the study of isozyme function is the phosphorylation-resistant GSK-3α/β knock-in mouse [43]. In these mice, GSK-3 could not be inhibited (via serine phosphorylation) by an upstream kinase. The results confirmed a dominant role for GSK-3β (but not GSK-3α) in regulating muscle glycogen synthase [43], as well as in vivo tau phosphorylation by GSK-3 [36]. These mice showed hyperactivity and mania, which recapitulated symptoms of schizophrenia and manic phase in bipolar disorder [44]; in another study, they showed impairment of neuronal precursor cell proliferation [45]. The recent development of isozyme selective GSK-3 inhibitors also provides an opportunity to distinguish differences in function between the two GSK-3 isozymes. The use of BRD0705 to selectively inhibit GSK-3α (IC_50_ 0.066 μM vs. 0.5 μM of α or β isoform respectively [46]), revealed that inhibition of GSK-3α corrects excessive protein synthesis and ameliorates the susceptibility to audiogenic seizures in Fragile X syndrome (FXS) mice [47]. Conversely, inhibition of GSK-3β by the selective inhibitor, BRD3731 (IC_50_ 0.015 μM vs. 0.215 μM of β or α isoform respectively [46]), reversed gamma oscillation and cognitive dysfunction in a mouse model of schizophrenia [32].

Overall, these findings suggest that GSK-3 activity is essential for normal neuron development, brain architecture, and “correct” behavior, while aberrant GSK-3 activity has deleterious effects on neuronal shape and brain functions. The GSK-3 isozymes exhibit both overlapping and distinct functions in the nervous systems. In some cases, there may be an absolute requirement for a given isozyme for a certain process, but in other cases, the activities of the two isoforms may be completely interchangeable. An absolute need for a certain isozyme is probably dependent on dosage, targeting, substrate, and timing.

## 2. GSK-3 in Neurodegeneration

GSK-3 is indeed a crucial player in the nervous system, and a significant factor that contributes to disease pathogenesis. Earlier studies revealed lithium salt, a drug approved for treating psychiatric disorders, as a GSK-3 inhibitor [48,49]. This finding implicated GSK-3 as a central regulator of mood behavior and psychiatric disorders, a notion that has since been supported by numerous studies. The current paradigm suggests that hyperactivity of GSK-3 is a causative factor in progressive neurodegenerative and psychiatric conditions, while inhibition of GSK-3 may be therapeutic. Indeed, hyperactive GSK-3 was found in the AD brain, and overexpression of GSK-3 in vivo induced AD pathology, cognitive deficits, and gliosis in a number of AD mice models [36,50,51,52,53,54,55]. Additional studies have reported that alterations in GSK-3 activity (e.g., either excessive activation, or inhibition) influence emotion, mood behavior, sociability skills, and schizophrenia-like behavior [31,33,44,56,57,58,59,60]. As a corollary, a reduction in GSK-3 activity reverses the severity of a number of diseases. For example, conditional deletion of GSK-3 in the brain of AD transgenic mouse models (mice expressing APP mutant, tau mutant, or double transgene expressing APP/PS1 mutants), was reported to reduce β-amyloid loads and levels of tau phosphorylation, and to decrease the formation of neurofibrillary tangles [30,61]. Likewise, treatment with GSK-3 inhibitors has been shown to improve disease symptoms in animal models of AD, Parkinson’s disease (PD), amyotrophic lateral sclerosis (ALS), Fragile X syndrome (FXS). Yet, no efficacy was achieved in phase 2 clinical trial for progressive supranuclear palsy (PSP) with the GSK-3 inhibitor tideglusib. Detailed descriptions of these studies have been published elsewhere [62,63,64,65,66,67].

**Figure 1:**
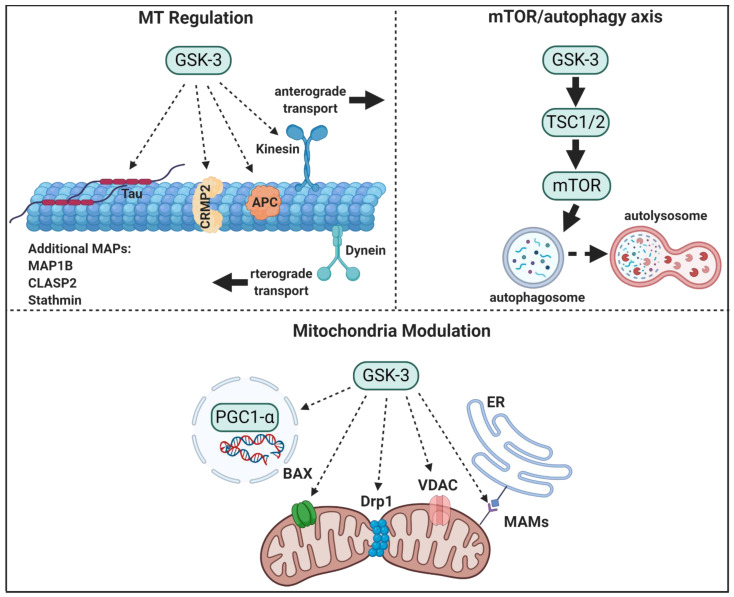
GSK-3 regulatory pathways in neurons. GSK-3 regulates microtubule (MT) stability and dynamics. Phosphorylation of MT binding proteins (MAPs) by GSK-3 reduces their binding to MT, and GSK-3 phosphorylation of kinesin 1 impairs anterograde and retrograde transport. GSK-3 activation of mTORC1 inhibits autophagic and lysosomal activity. GSK3 regulates mitochondrial energy metabolism and mitochondria-mediated cell death. GSK-3 destabilizes peroxisome proliferator-activated receptor γ, PGC1α, and inhibits its transcriptional activity, phosphorylation of dynamin-related protein1, DRP1, by GSK3 enhances mitochondria fission, and phosphorylation of Voltage-dependent anion-selective channel 1, VADC1, and bcl-2 associated proteins, Bax, by GSK-3 enhances their induced-apoptotic activity. Finally, GSK-3 impairs mitochondria and ER communication by disrupting proteins associated with the microdomain, mitochondria-associated membranes, MAM.

It is evident from the accumulated data that GSK-3 plays a prominent role in regulating structural and metabolic processes both in developing and adult neurons. In this review, we describe the role of GSK-3 in regulating cytoskeleton organization, the mammalian target of rapamycin (mTOR) pathway, and in mitochondria, all of which are components that link GSK-3 to neurodegeneration (see Figure 1). In addition, we provide an update of the field of GSK-3 inhibitors.

## 3. GSK-3—A Regulator of Microtubule Network and Axonal Transport

Cytoskeletal dysfunctions have been implicated in a variety of neurodegenerative diseases. Microtubule (MT) assembly is important in providing a major building block of the cytoskeleton, and, through polymerization and bundling, MTs influence neuronal- shape and polarity, axon growth, transportation, and migration [68,69,70]. The microtubule plus ends display “dynamic instability,” a cycling switch between rapid growth (polymerization) and shrinkage (de-polymerization) that enables MTs to direct neuron growth and regulate neuronal network wiring. MTs also serve as tracks for the motor proteins such as the kinesins and dyneins, which transport molecules and vesicles across the soma to axon endpoints. Indeed, alterations in MT dynamics are associated with early stages of neurodegenerative progression in diseases such as AD, PD, Huntington’s disease (HD), Charcot-Marie-Tooth (CMT), and ALS [71,72]. The ability of GSK-3 to regulate axon morphogenesis and axon transport relies on its ability to regulate number of MT-binding proteins (MAPs) and motor proteins. These include MAP1B [73], tau [74], collapsin response mediator proteins, (CRMPs) [75], adenomatous polyposis coli (APC) [76,77], cytoplasmic linker associated protein 2 (CLASP2), and the MT-depolymerizing factor stathmin [78], also reviewed in [79], and kinesin 1 [80,81,82]. Phosphorylation of MAPs by GSK-3 abolishes their binding capacity for MTs, thus, disrupting “correct” MT assembly and reducing stability. In addition, GSK-3 regulates the motor proteins and can impact cargo trafficking. Not surprisingly, GSK-3 has been recognized as a master regulator of neuronal development and morphogenesis [40,83] (also described in previous section). The role of GSK-3 as a “tau kinase” is perhaps the most “famous” link between GSK-3 and AD [84,85,86]. Tau serves as an MT stabilizer, and omitting tau from MTs results in structural defects of synapses, axons, and dendrites [87,88]. GSK-3 phosphorylates tau at many sites in vitro [85]; however, a limited number of sites were found to be phosphorylated in cells and tissues, these include the sequence stretch of Ser 396, Ser 400, and Ser 404, where Ser 404 is the priming site phosphorylated by CDK-5, and Thr 231, Thr 235 (AT180 epitope) [19,89]. Phosphorylation of tau by GSK-3 reduces tau binding to MTs [90] and it then accumulates as insoluble tau aggregates that form neurofibrillary tangles (NFTs). These are a major cause of neuron degeneration and dementia in AD and other tauopathies [91,92]. The combined activities of GSK-3 and CDK-5 can produce tau hyper-phosphorylation in the brain [42,93], and increased activity of CDK-5 is also sufficient to induce NFT formation and MT destabilization due to its activity as the priming kinase [94,95].

GSK-3-tau phosphorylation was demonstrated to be an important event in neurodegeneration: Activation of GSK-3 by β-amyloid intensifies neuronal tauopathy in the mouse brain [53], although in tau-knockout mice, GSK-3 was unable to induce hippocampal degeneration and learning deficits [96]. Loss of both GSK-3α and β reduced tau phosphorylation in transgenic mice [30], and the use of GSK-3 inhibitors further confirmed that reducing tau phosphorylation through inhibition of GSK-3 is sufficient to provided neuroprotection [97,98]. Since hyper-phosphorylation of tau is also found in PD and HD [99,100,101], GSK-3 may serve as a tau kinase in these diseases. Indeed, inhibition of GSK-3 was shown to alleviate the accumulation of α-synuclein and mutant huntingtin (mHtt) by reducing tau phosphorylation [102].

APC is a component of the Wnt signaling-APC/Axin destruction complex [103,104] and a well-established tumor suppressor in human colorectal cancer [105]. The protein belongs to the group of “plus end tracking proteins” (+TIPS) that promote MT plus end polymerization, which has a profound impact on neurite extension, cell migration, and neuronal polarity [106,107,108,109]. Localization of APC at the tips of the prospective axon is required for correct targeting of the polarity kinase, PAR3, to the axonal growth cone, where it interacts with protein partners to establish neuronal growth and polarity [77]. GSK-3β binding to APC and promoting APC phosphorylation [103,110] was shown to reduce MT stability [111], and to disturb neuronal polarity at the nascent axon tip [77]. In contrast, inhibition of GSK-3 by PAR4 increased APC binding to MT and promoted neuronal migration in the developing neocortex [112]. Similarly, inhibition of GSK-3 by the nerve growth factor, NGF, reduced APC phosphorylation and APC promoted axon cone growth [76].

The collapsin response mediator proteins (CRMPs) promote MT assembly and facilitate MT polymerization. Their involvement in neurodegenerative disorders is now increasingly recognized [113,114,115]. CRMP2 protein, the most studied of the five family members, is implicated in axon elongation, branching, and specification [39]. The protein is highly expressed in developing neurons, while lower levels of CRMP2 are sufficient to retain differentiation and plasticity in the adult neurons. MT polymerization is mediated by the interaction of CRMP2 with the tubulin heterodimers in a process that enhances the GTPase activity of the β-tubulin subunit [116,117]. CRMP2 also serves as a negative regulator of semaphorin 3A (Sema3A), a neurite retracting factor. Phosphorylation of CRMP2 by GSK-3 attenuates MT polymerization and enables Sema3A induced growth cone collapse [118,119]. The GSK-3 phosphorylation sites on CRMP2 were identified as Thr 509-Thr 514-Ser 518, and the priming site as Ser 522 phosphorylated by CDK-5 [75,118,120]. Phosphorylation of CRMP2 by GSK-3 is also implicated in axon degeneration of dopaminergic neurons [121], NFT formation [118,120], impaired neuronal polarity [75], and reduced cognitive functions [122]. Since de-phosphorylation of CRMP2 has been recognized as a therapeutic approach, the use of GSK-3 inhibitors as reagents that cargo and MTs. Phosphorylation of KCL2 by GSK-3 reduces kinesin-1 binding to MTs and also dissociates the protein from the cargo motor complex [80,81,82]. Defective transport may be related to pathogenesis, for example impaired APP transport by GSK-3 has been shown to cause premature amyloid plaque deposition [72,123]. These studies also demonstrated an additional mechanism of GSK-3 in reducing the number of active kinesin (in addition to reduced MT/cargo binding) [123]. Thus, decreased axonal transport of APP could be an early event in AD progression by increasing plaque deposition [123]. Conversely, inhibition of GSK-3 would stimulate APP transport and presumably enhance synaptic growth and function [124]. Similarly, hyperactive GSK-3 reduced the transport of synaptophysin- and syntaxin-I that are important for synaptic growth [81]. Behavior may also be affected by defective transport as demonstrated by the depressive and manic behavior seen after impaired transport of GluR1/kinesin1complex induced by GSK-3-phosphorylation of KCL2 [59].

Dynein is not directly phosphorylated by GSK-3; however, since both motor proteins are required for cargo transport, dynein motor ability is directly affected by the phosphorylation of kinesin1 by GSK-3 [123,125,126].

As a last caveat, GSK-3 regulates transport indirectly, via phosphorylation of CRMP2. This is due to the fact that CRMP2 interacts with the KLC1 [127], and enables transport of downregulate phosphorylation at CRMP2 is considered of therapeutic value [120]. An interesting clinical indication has been described for certain CNS injuries where a reduction in CRMP2 phosphorylation due to GSK-3 inhibition induced axon regeneration in the injured spinal cord and optical nerve [128,129]. Reduced CRMP2 phosphorylation as a result of GSK-3 inhibition has also been implicated in reversing axon degeneration of dopaminergic neurons [121], limiting axonopathy in multiple sclerosis [130,131], and attenuating β-amyloid-induced cognitive dysfunction in AD [132].

GSK-3 could also be a regulating factor in ALS through the ability to phosphorylate CRMP4 [16], a protein that was recently implicated in ALS. Like CRMP2, CRMP4 regulates growth cone dynamics [133], and aberrant expression of CRMP4 induces neuromuscular degeneration and motor neuron death [134,135]. It is possible that the damage caused to motor neurons by CRMP4 could be mediated by phosphorylation by GSK-3, although this scenario will require further validation.

Axon transport of essential biological cargo from the cell body to the axon tip is essential for neuronal survival and failure of this process is indeed a critical event in neuron degeneration [136,137,138,139,140]. Transport is mediated by the motor proteins kinesins and cytoplasmic dyneins, which generate force and movement along the microtubules by increasing the number of motor proteins associated with the moving cargo, or, by generating multimeric motor complexes [141]. The kinesin superfamily are MT plus end-directed and mediate anterograde transport toward the synapse, while dyneins mediate retrograde transport of the MT minus end-directed from the axon tips to the soma [141]. Kinesin-1 is the best studied member of the kinesin family and is composed of two kinesin heavy chain (KHC) and two kinesin light chain (KLC) subunits that participate in binding components such as tubulin and WAVE1 Wiskott-Aldrich syndrome protein family verprolin-homologous protein [114,127]. Phosphorylation of CRMP2 releases the CRMP2/kinesin1 complex and impairs their anterograde transport [142].

In summary, GSK-3 plays a central role in regulating MT stability, polymerization, and dynamics, and MT-mediated axon transport (Figure 1). Moreover, GSK-3 regulation of axon transport has important clinical implications as failure of cargo delivery into to neuron tips had been implicated as an early event in a number of neurodegenerative disorders [72,136].

## 4. GSK-3—An Upstream Regulator of mTORC1/Autophagy Axis

The mammalian target of rapamycin, mTOR, is a crucial nutrient-sensing hub protein that mediates growth factor signaling and coordinates the metabolic response [143,144,145,146]. mTOR is found in two multimetric complexes, namely mTORC1 and mTORC2, which are distinguished by a unique accessory protein (raptor or rictor respectively), and by their differential sensitivity to rapamycin [143]. mTORC1 is a master growth regulator that is stimulated by nutrients and growth factors to upregulate the synthesis of cellular building blocks (proteins, lipids, DNA). This anabolic activity of mTORC1 is coordinated by the suppression of autophagy, which serves as a main pathway for sequestration and targeting cytoplasmic components to the lysosome [147,148,149,150,151]. Inhibition of mTORC1 in response to stress or starvation facilitates the formation of new autophagosomes that recruit cellular waste and carry the cargo to lysosomes. [152,153,154]. Inhibition of mTORC1 also triggers lysosomal biogenesis by activating the transcription factor EB (TFEB), and the lysosomal acidification that is required for optimal function of lysosomes [155]. In addition, mTORC1 activity is interrelated with that of AMP-activated protein kinase (AMPK), a principal sensor for energy stressors, which activates autophagy upon stress or nutrient starvation. Hence, the “correct” coordination of mTORC1 and AMPK activity is essential for balanced cellular homeostasis [156].

Deregulation of mTORC1 had been implicated in numerous examples of brain dysfunction including mental retardation, aberrant behavior, and impaired brain development [157,158,159,160]. Furthermore, impaired autophagy has been implicated in aging and neurodegeneration: lack of ATG7, a gene essential for autophagosome formation, resulted in an accumulation of inclusion bodies that was associated with massive neuronal loss [161], while the deletion of Beclin 1, a protein required for autophagy, increased the accumulation of intraneuronal β-amyloid associated with neuronal ultrastructural abnormalities. Dysregulation of autophagy also enhanced the accumulation of α-synuclein in the PD brain [162,163]. A failure in autophagosomes-cargo recognition increased the levels of mHtt in cells [164], while activation of autophagy accelerated the clearance of β-amyloid, α-synuclein, and mHtt [165,166,167,168,169], provided neuroprotection, and supported longevity [168,169,170]. Thus, finding agents that activate autophagy is considered a promising approach for treating neurodegenerative diseases [152,171,172,173,174,175,176].

GSK-3 has been implicated as an upstream regulator of mTORC1, autophagy, and lysosomal acidification (Figure 1). Earlier studies identified the protein translational factors eukaryotic initiation factor-2 (eIF2B) and eukaryotic elongation factor-2 (eEF-2), as cellular downstream targets of GSK-3 [177,178]. In addition, the results indicated that GSK-3 regulates the negative regulator of mTORC1, Tuberous Sclerosis Complex, TSC1/2 [179,180]. Phosphorylation of TSC2 by GSK-3 was coordinated with AMPK that serves as the “priming kinase” and inhibits TSC1/2 activity [181]. Although this suggests that GSK-3 is a negative regulator of mTORC1 [180], there are other reports that GSK-3 is rather a positive regulator of mTORC1. Overexpression of GSK-3α/β in cells resulted in activation of both mTORC1 [182,183] and the mTORC1 target, S6 ribosomal kinase (S6K-1) [184]. In addition, GSK-3 was shown to inhibit AMPK and thus could prevent the inhibition of TSC2 by AMPK [185]. Interestingly, ablation of AMPK phosphorylation by GSK-3 resulted in constitutive autophagic activity and inability of cells to respond to anabolic conditions [185]. Genetic studies in yeast further indicated for a potential role of AMPK/GSK-3 axis. Under glucose starvation conditions, AMPK and MDCK-1 (the yeast GSK-3 ortholog) cooperated into the metabolic response programing to promote mitochondria respiration and proteostasis maintaining that eventually supported extended longevity [186].

Taken together, it seems clear that inhibition of GSK-3 activates autophagy via inhibition of mTORC1. Studies with GSK-3 inhibitors indeed confirmed this paradigm by demonstrating that treatment with GSK-3 inhibitors inhibited mTORC1, increased autophagic activity and triggered lysosomal biogenesis and acidification [182,187,188,189]. The GSK-3/mTORC1 axis is indeed an important avenue in neuronal signaling and has been implicated in synaptogenesis and axonal repair [190], mood behavior [191], aging, and longevity [192,193]. It is noteworthy that GSK3α KO mice exhibit shortened lifespans and increased age-related pathologies that can be rescued by inhibition of mTORC1 [28]. Finally, GSK3 has recently been implicated as a negative regulator of mTORC2 as a result of phosphorylating the accessory protein Rictor and triggering its proteasomal degradation [194]. Resolving the GSK-3/mTORC2 autophagy paradigm will require further study, but could explain the therapeutic activity achieved with GSK-3 inhibitors as neuroprotective and anti-aging drugs. Their ability to restore impaired autophagic/lysosomal activity and to promote the clearance of toxic proteins is of great advantage in treating neurodegenerative disorders.

## 5. GSK-3 and Mitochondria—Energetic Regulation and Cell Death

Mitochondria are essential for neuronal function, providing the energy required to power neurotransmission and fulfilling many other physiological roles. In neurons, mitochondria should be efficiently transported to synapses and other sites, where their functions are required. Thus, neurons, with their highly elongated morphology, are extremely sensitive to local mitochondria delivery of ATP. Correct mitochondrial traffic is critical to ensure proper position of mitochondria to serve the metabolic needs of neurons. How does mitochondria contribute to neurodegeneration? Impaired oxidative phosphorylation could be certainly one of the causes contributing to neurological conditions [195], in addition, a growing notion in the filed considers mitochondria as an integrated subcellular system and suggests that defects in mitochondria dynamics is the dominant factor that contributes to the pathogenesis of neurodegenerative disorders [196]. These defects include aberrant mitochondria trafficking, impaired interorganelles communication, defects in mitochondria quality control and impaired mitophagy, all play a central role in the pathogenesis of neurodegenerative disorders [196,197].

A major mitochondria GSK-3 target is the peroxisome proliferator-activated receptor γ coactivator 1-alpha, PGC-1α, a transcriptional co-activator that regulates many aspects of mitochondrial functions. Activation of PGC-1α increases the expression of target genes coincident with increased mitochondria respiration and biogenesis. GSK-3 destabilizes PGC-1α by targeting the protein to intranuclear proteasomal degradation [198]. Degradation of PGC-1α was initiated by GSK-3 phosphorylation (at Thr-295) that enables recognition by E3 ubiquitin ligase that triggers ubiquitin mediated proteolysis [199]. In vivo studies indeed confirmed that inhibition of GSK-3 (by lithium) resulted in upregulation of PGC-1α and accompanied with elevation in cytochrome c oxidase and increased mitochondria respiration in the mouse hippocampus [200].

The final step in mitochondrial fission process is executed by the Dynamin-related protein1 (Drp1), which facilitates membrane splitting via constriction and GTPase activity. GSK-3 phosphorylates Drp1 (at Ser^40^ and Ser^44^) that activates GTPase activity of Drp1 leading to mitochondrial fragmentation that exacerbates pro-apoptotic activity [201].

GSK3 phosphorylates the voltage-dependent anion channel 1, VDAC1, an abundant protein located at the outer mitochondrial membrane. Phosphorylation of VADC1 blocks its interaction with hexokinase II and subsequently disrupts the anti-apoptotic function of hexokinase II [202]. In addition, the interaction of GSK-3 with VDAC2 increases the mitochondrial permeability transition pore (mPTP), a principle trigger of cell death, and enhances oxidative stress-induced cell death [203]. An additional connection with mitochondria-mediated cell death pathway is GSK-3 regulation of the bcl-2 family member, Bax. In this scenario, GSK-3 phosphorylates Bax (ar Ser 163), promotes Bax translocation into the mitochondria where it induces its pro-apoptotic activity via the mitochondria death pathway [204].

ER and mitochondria have a continuous interaction that maintains “appropriate” intracellular homeostasis. This communication is achieved by a physical association between the two organelles which is formed by a specific microdomain termed MAMs: mitochondria-associated membranes [205]. GSK-3 reduces this ER-mitochondria connection platform by reducing the association of proteins interacting with MAMs this in turn, impairs calcium and phospholipid exchange as well as ATP production and ER stress response activity [206].

It is noteworthy that different conclusions were reported regarding GSK-3 regulation of mitochondria transport. For example, overexpression of GSK-3β resulted in increased mitochondria motility in hippocampal neurons, suggesting that inhibition of GSK-3 rather inhibits mitochondria axonal transport [207]. On the other hand, excess of GSK-3β activity in PC12 cells or in cortical neurons decreased mitochondria anterograde transport, and increased pausing periods [208], while inhibition of GSK-3 stimulated mitochondria movement [209]. It appears that these differences largely depend on the type of MAPs phosphorylated by GSK-3. Thus, GSK-3 induced phosphorylation of tau, MAPB1 or kinesin 1, results in a different impact on mitochondria movement that is likely dependent on cell type and gradient alterations in GSK-3 expression [207,208,210].

Taken together, GSK-3 disrupts cellular homeostasis and regulates energy production by impairing mitochondria functions and promoting mitochondria-mediated death pathways (Figure 1).

## 6. GSK-3–A Target for Inhibition

The number of GSK-3 inhibitors is continuously rising and many diverse chemotypes from cations to natural products, peptides, and small molecules have been tested in cellular and physiological systems [62,64,211]. The best characterized GSK-3 inhibitors are ATP competitive inhibitors, which target the ATP binding site of the kinase. The problem with ATP competitive inhibitors is that their specificity is limited by the fact the ATP binding site is highly conserved across the protein kinase family [212,213]. Another problem with this type of inhibitors is their tendency to induce drug resistance, mainly as a result of the formation of point mutations at the ATP binding site. A well-known example is the drug resistance developed by patients treated with imatinib, an ATP competitive inhibitor of the oncogene BCR-Abl1 and an FDA approved drug for treating chronic myeloid leukemia (CML) [214]. Indeed, the severe side effects of the inhibitors that were tested to date have resulted in very few being investigated in clinical trials. Another issue with the ATP competitive inhibitors is that they do not discriminate between the GSK-3 isoforms. As described earlier, GSK-3 isozymes may play distinct and individual roles in controlling cellular process, and therefore, it may be of significant value to selectively inhibit only a specific isozyme. One study initially reported a novel scorpion-shaped molecule with high selectivity towards GSK-3α [215], and improved derivates of this molecule exhibited anti-cancer activity in treating acute myeloid leukemia (AML) [216]. A different study exploited the Asp133-Glu196 switch in the hinge-binding region between the GSK-3 isozymes to develop selective GSK-3α inhibitors with therapeutic activity in treating AML and FXS [46,47]. A possible approach to resolve this issue is to search for repurposing molecules whose toxicity has been already tested, as GSK-3 inhibitors. Recently, a machine learning based algorithm was used to virtually screen FDA approved drugs [217] and identified ruboxistaurin, an anti-diabetes drug, as a GSK-3 inhibitor. The machine learning model also attempted to predict the selectivity of compounds toward GSK-3 isozymes and classified ruboxistaurin as a selective GSK3β inhibitor [217].

Developing inhibitors that do not target the ATP binding site may be an advantage in generating suitable drugs. A non-ATP competitive inhibitor, of the thiadiazolidinone class, termed “tideglusib,” was developed for treating AD [218]. It was suggested to act as an allosteric inhibitor [219], and to bind to the inactive conformation (“DFG-out”) of GSK-3 [220]. Tideglusib entered the clinical phase; however, the trial was reported to have missed its primary endpoint in treating AD or Progressive Supranuclear Palsy (PSP) [66,67]. Additional clinical trials tested the therapeutic activity of tideglusib in autism and myotonic dystrophy (see also clinicaltrials.gov) [221,222]. Substrate competitive inhibitors (SCIs) may also represent a possible solution to the problem of specificity. Because the substrate binding site is less conserved among protein kinases (and, consequently, more specific), SCIs are expected to show higher selectivity than the ATP analogues. However, they are also expected to show weak inhibition as the binding affinity to the substrate is relatively weak, and this consideration has so far precluded the development of this type of inhibitor. However, strong constitutive inhibition of protein kinases in the biological system, is now recognized as likely to be harmful, and moderate inhibition of the kinase may be preferable for long-term treatment. This is particularly true in the case of GSK-3 as an enzyme whose activity is essential for the normal function of neurons. Furthermore, the “pathological” levels of GSK-3 activity do not exceed 2- to 3-fold “normal” levels. Thus, moderate-to-weak inhibition of GSK-3 (about 50%) is now actually recommended for treating conditions associated with elevated levels of GSK-3 activity. The development of efficient SCIs is challenging because the ambiguous, non-well-defined nature of the substrate binding site makes it a difficult target for drug design. In addition, high-throughput screening often identifies molecules that are “trapped” in the small defined pocket of the ATP binding site. Fortunately, the advanced computational and screening tools developed over the past decade have tremendously improved our ability to discover and design target-based molecules as potential drugs.

**Figure 2:**
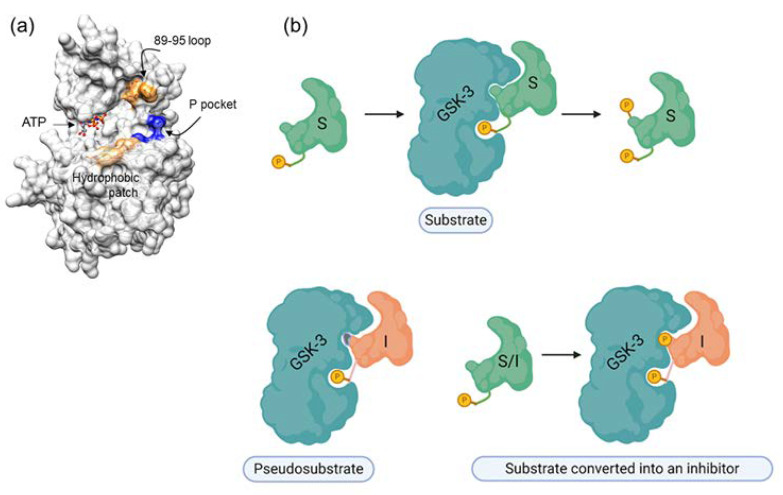
GSK-3 SCIs. (**a**) GSK-3 substrate binding sites are highlighted. Blue, the phosphate binding pocket; orange, the 89-95 loop; beige, the hydrophobic patch (Ile, 213, Val 214, Tyr 216). (**b**) Types of substrate competitive inhibitors (SCIs): the primed substrate is phosphorylated by GSK-3 and upon phosphorylation dissociates from the enzyme, the pseudosubstrate SCI is a mutated substrate that cannot be phosphorylated by the kinase, the “substrate converted into an inhibitor” is a substrate that upon phosphorylation remains bound to the kinase.

Our rationale for developing SCIs for GSK-3 was based on the unique substrate recognition motif, which involves a phosphorylated residue (serine or threonine) arranged as SXXXS(p) (where X is any amino acid and S(p) is a phosphorylated serine). It was therefore reasonable to assume that short peptides with this pattern might serve as adequate inhibitors. In addition, a thorough understanding of the binding mode of the substrate or the “prototype” peptide inhibitor could enable us to improve the inhibitory capabilities of SCI inhibitors. The GSK-3 substrate binding site includes a segment termed the “89–95” loop, which is bordered by Gln 89-Asn 95, and in which Phe 93 is the most critical residue for binding [223,224]. In some cases, an additional “hydrophobic patch” (Val 214, Ile 217, and Tyr 216) is also involved in substrate binding [223,224]. The resultant peptide inhibitors are of the pseudosubstrate inhibitor type (Figure 2).

Surprisingly, we discovered a unique in situ substrate-inhibitor conversion mechanism, in which the inhibitor acts as a substrate, but, once phosphorylated, becomes an effective inhibitor [225]. This is apparently because phosphorylation of the “substrate-inhibitor” by GSK-3 enhances the affinity for the substrate binding site as a result of the interaction between the two phosphorylated groups and the phosphate binding pocket [225]. The therapeutic potential of the SCI peptides has now been demonstrated in a number of cellular and disease mouse models including Parkinson’s, Alzheimer’s disease, multiple sclerosis, depressive behavior, and Fragile X syndrome [56,187,225,226,227,228,229]. Recently, we took a further step and developed novel GSK-3 SCI small molecules that mimicked the integration of SCI peptides with the kinase [230].

The available FDA-approved treatments that are designed to slow neurodegeneration do not stop or slow the disease progress, and there have been no new treatments approved in the last decade. There is therefore a desperate need for new treatments. GSK-3 inhibitors have demonstrated efficacy in multiple disease models and it is expected that this approach will prove fruitful in clinical trials. The problem of safety in the drug development process may be overcome by the use of different types of inhibitor to those that are commonly used at present. This may provide a new hope for future treatment.

## 7. Conclusions

From its initial identification as a regulator of glycogen metabolism, GSK-3 has been implicated in numerous fundamental pathways. Important insights, however, now suggest that GSK-3 is a dominant regulator in brain functions and it has been shown to increase neuron damage and disease vulnerability. Growing evidence uncovered distinct roles for each of the GSK-3 isozymes along with other studies that found redundancy or compensatory activity between the two isozymes. Taking into consideration that the evolution of GSK-3α differs dramatically from that of GSK-3β, it is important to deepen our understanding of the unique biochemical, structural, and biological properties of GSK-3α, the isozyme that has been “historically” overlooked (as compared to GSK-3β). Certainly, some clues could be extracted from the GSK-3 knockout systems together with the studies that used selective isozyme inhibitors. They further brought into our attention and recognition that our understanding of the dynamics and cross-talk of the two isozymes is not complete. If hyperactivity of GSK-3 is indeed a causative factor of disease pathogenesis, the development of potent GSK-3 inhibitors could represent an excellent therapeutic strategy. Does selective inhibition of α/β isozyme is a better approach in treating neurodegeneration as compared to the use of pan GSK-3 inhibitors? The GSK-3 knockout (postnatal) systems did not suggest beneficial outcomes produced by abolishing the activity of one isozyme over the other. On the other hand, selective inhibition of GSK-3α showed potential efficacy in treating Fragile X syndrome [47]. We believe, however, that inhibition of both isozymes may be a favorable approach. Excessive inhibition of a specific GSK-3 isozyme may be toxic to neurons, and in addition, inhibition of one isozyme may be compensated by the other (either by increased activity or by elevated expression levels of the “non-inhibited” isozyme). Thus, maintenance of exquisite balance of GSK-3 activity by inhibiting both isozymes may be a better approach for clinical practice.

A major hurdle that should be taken into consideration in this respect is the inability of most drugs to cross the blood-brain barrier (BBB). This barrier formed by extremely tight junctions between connected endothelial cells of the brain microvessels, hinders the access of small molecules, peptides, or proteins into the brain. However, in recent years various drug delivery tactics were developed to assist drugs to penetrate the brain. These include chemical modification of pro-drugs, use of liposomes, and the use of polymeric nanoparticles as drug carriers [231,232]. These new approaches will provide satisfactory solutions for effective CNS therapeutics in the future.

Unfortunately, to date, only a few GSK-3 inhibitors have been found suitable to enter the clinical phase. Our suggestion is to use a new type of inhibitors with a unique inhibition modality that is expected to be more successful in clinical practice. It is hoped that such novel GSK-3 inhibitors will provide new opportunities for treating neurodegenerative disorders.

## Data Availability

Not applicable.
